# CD4 T-Cell Subsets and the Pathophysiology of Inflammatory Bowel Disease

**DOI:** 10.3390/ijms24032696

**Published:** 2023-01-31

**Authors:** Raquel Gomez-Bris, Angela Saez, Beatriz Herrero-Fernandez, Cristina Rius, Hector Sanchez-Martinez, Jose M. Gonzalez-Granado

**Affiliations:** 1 LamImSys Lab, Instituto de Investigación Sanitaria Hospital 12 de Octubre (imas12), 28041 Madrid, Spain; 2 Departamento de Fisiología, Facultad de Medicina, Universidad Autónoma de Madrid (UAM), 28029 Madrid, Spain; 3 Facultad de Ciencias Experimentales, Universidad Francisco de Vitoria (UFV), 28223 Pozuelo de Alarcón, Spain; 4 Department of History of Science and Information Science, School of Medicine and Dentistry, University of Valencia, 46010 Valencia, Spain; 5 UISYS Research Unit, University of Valencia, 46010 Valencia, Spain; 6 CIBER de Enfermedades Cardiovasculares (CIBERCV), 28029 Madrid, Spain; 7 Department of Immunology, Ophthalmology and ENT, School of Medicine, Universidad Complutense de Madrid, 28040 Madrid, Spain; 8 Centro Nacional de Investigaciones Cardiovasculares (CNIC), 28029 Madrid, Spain

**Keywords:** adaptive immune system, inflammatory bowel disease, ulcerative colitis, Crohn’s disease, Th1, Th2, Th17, Th19, Th22, regulatory T-cell, Treg

## Abstract

Inflammatory bowel disease (IBD) is an umbrella term for the chronic immune-mediated idiopathic inflammation of the gastrointestinal tract, manifesting as Crohn’s disease (CD) or ulcerative colitis (UC). IBD is characterized by exacerbated innate and adaptive immunity in the gut in association with microbiota dysbiosis and the disruption of the intestinal barrier, resulting in increased bacterial exposure. In response to signals from microorganisms and damaged tissue, innate immune cells produce inflammatory cytokines and factors that stimulate T and B cells of the adaptive immune system, and a prominent characteristic of IBD patients is the accumulation of inflammatory T-cells and their proinflammatory-associated cytokines in intestinal tissue. Upon antigen recognition and activation, CD4 T-cells differentiate towards a range of distinct phenotypes: T helper(h)1, Th2, Th9, Th17, Th22, T follicular helper (Tfh), and several types of T-regulatory cells (Treg). T-cells are generated according to and adapt to microenvironmental conditions and participate in a complex network of interactions among other immune cells that modulate the further progression of IBD. This review examines the role of the CD4 T-cells most relevant to IBD, highlighting how these cells adapt to the environment and interact with other cell populations to promote or inhibit the development of IBD.

## 1. Introduction

The immune system is divided into two main branches, the innate and adaptive immune responses. Innate immune cells, which include neutrophils, monocytes, macrophages, and dendritic cells (DCs), respond rapidly and non-specifically to pathogens or other foreign entities as a first line of defense. Innate immune cells express pattern recognition receptors (PRRs), such as Toll-like receptors (TLRs) and NOD-like receptors (NLR), that allow them to recognize pathogen-associated molecular patterns (PAMPs) and damage-associated molecular patterns (DAMPs), provoking their activation. Once activated, innate immune cells provoke inflammation by releasing cytokines and chemokines, activating the complement cascade and phagocytosing pathogens and cell debris. Some innate immune cells take up, process, and present antigens to activate the adaptive immune response, acting as antigen-presenting cells (APCs). Adaptive immunity depends on this antigen presentation by APCs, and the cytokine milieu generated by the innate response and thus takes longer to activate than innate immunity, but the corollary is that adaptive immunity is highly specific. The key cells of the adaptive immune system are CD4 and CD8 T-cells and B cells. Natural killer T-cells (NKT cells) and γδ T-cells are cytotoxic T lymphocytes that sit at the boundary between innate and adaptive immunity [1]. Profuse data show that the innate and adaptive immune systems both play significant roles in the origin and development of IBD [2,3,4,5,6,7,8,9,10].

IBD is a chronic immune-mediated idiopathic inflammation of the gastrointestinal tract with a prolonged period of relapse and remission [11,12,13,14]. Worldwide, 6.8 million people suffer from IBD [15]. IBD is considered a global disease, and its evolution can be stratified into four epidemiological stages, including emergence, acceleration in incidence, compounding prevalence, and prevalence equilibrium. Developing countries and newly industrialized countries are in the emergence and the acceleration in incidence stages, respectively, and Western regions are in the compounding prevalence stage and will eventually transition to the prevalence equilibrium stage [15]. Despite the success of IBD therapy, patients have a mortality risk 1.5 times higher than that of the healthy population, and mortality linked to IBD continues to increase progressively [16]. The stages of IBD range from mild to moderate to severe [17,18]. The main clinical manifestations of IBD are Crohn’s disease (CD) and ulcerative colitis (UC). These diseases differ in some symptoms, disease location, and histopathological characteristics but share gastrointestinal symptoms such as diarrhea, mucus, and bloody stools, and abdominal pain, as well as extraintestinal symptoms, such as arthritis, oral ulcers, skin lesions, and ophthalmological problems [19,20,21,22]. The numerous IBD complications include strictures, abscesses, fistulas, and colitis-associated cancer [23].

Even though the pathophysiology of IBD has not been fully defined, the etiology of the disease is known to involve a combination of genetic, environmental, microbiological, and immunological factors that promote intestinal barrier dysfunction and tissue damage, and dysregulated innate and adaptive immune responses [13,24,25].

A key feature in the appearance, progression, and prognosis of IBD is an aberrant intestinal mucosal immune system, and a common disease denominator in all IBD patients is the infiltration of intestinal tissue by inflammatory T-cells [26,27] and the accumulation of several proinflammatory cytokines associated with activated T-cells [28,29,30]. 

T-cells are classified broadly into proinflammatory and anti-inflammatory populations that form three main groups. The proinflammatory CD8 T-cells have cytotoxic capacity and are implicated in the response to tumors, metastatic cells, and viral infections [31]. CD4 T helper (Th) cells regulate the inflammatory milieu, promoting antibody production, controlling innate immunity, and stimulating immunologic memory. The third category is an anti-inflammatory CD4+ population called regulatory T-cells (Tregs), which suppress inflammatory responses, promote immunological tolerance, and control immune responses to prevent autoimmunity [32,33].

**Figure 1:**
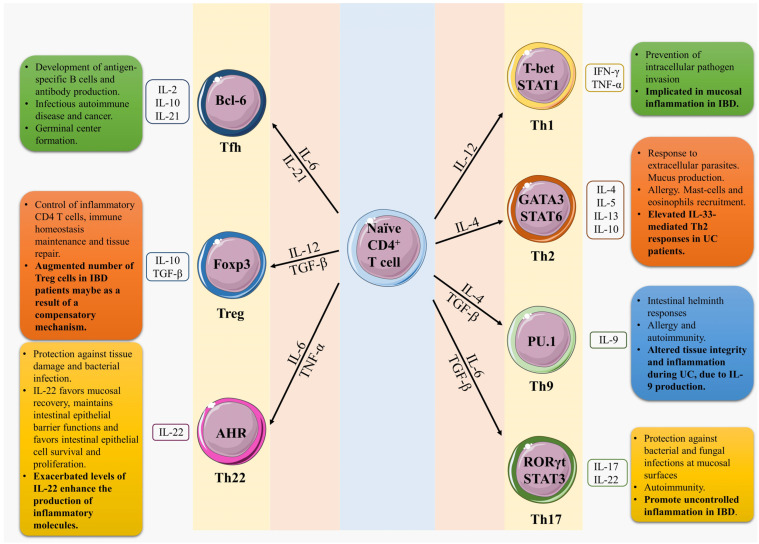
T-cell differentiation, subsets, and main functions. Naive CD4 T-cells can undergo differentiation into distinct effector subsets (e.g., Th1, Th2, Th9, Th17, and Th22 cells), follicular helper T (Tfh) cells, and regulatory phenotypes (Treg), each producing a characteristic set of cytokines (unfilled boxes next to cells). This differentiation process is mediated in part by the local cytokine microenvironment (arrows), which activates specific transcription factors and signaling molecules (text inside cells). Color-filled boxes next to cells list functions in homeostasis and in IBD (bold text). IBD is associated with changes in T-cell populations.

With the exception of natural Tregs, which are generated in the thymus during the positive selection of maturing T-cells [32], post-thymic naïve CD4 T-cells are nonactivated T-cells that have no contact with their cognate antigen and retain multiple differentiation capabilities [34]. Naïve T-cells undergo a functional and transcriptional programming called differentiation upon the recognition of an antigen presented by an APC in a secondary lymphoid organ. This recognition involves the binding of T-cell antigen receptors (TCRs) to host major histocompatibility complex (MHC) molecules complexed with foreign peptides [35]. After antigen recognition and the formation of an immune synapse with the APC [36], naïve CD4 T-cells are activated and undergo clonal expansion before differentiation. Changes in CD4 T-cells are mediated by a variety of factors, including the strength of the TCR signal, the cytokine microenvironment, and co-stimulation by the APC. These changes include chromatin remodeling and the modification of DNA methylation and promote the activation or suppression of specific transcription factors that direct the differentiation towards at least seven distinct T helper cell subsets: Th1, Th2, Th9, Th17, Th22, T follicular helper (Tfh), and several types of Tregs [37,38,39]. These CD4 T-cell subsets are classified according to their cytokine production and expression of master transcription factors [40]. T-cell subsets are further distinguished by the production of different components of the signaling transducer and activator (STAT) family (Figure 1).

Detailed knowledge of the functions of these lymphocyte subpopulations is essential for defining the complicated molecular and cellular pathways underlying IBD. In the following sections, we outline the main characteristics of these CD4 T-cell subsets and their positive or negative influence in IBD onset and progression.

## 2. T-Cells in IBD

Under steady state conditions, the gut contains scattered interepithelial lymphocytes and innate lymphocytes in the epithelial layer of the intestinal mucosa, with very few CD4 T-cells [41]. In contrast, IBD is associated with an abundance of CD4 T-cells in the epithelial layer of the inflamed intestinal mucosa [42] or with normal numbers of lamina propria and epithelial CD4 T-cells [43,44] but showing increased activation [45,46,47] and phenotypic alterations [48].

T-cells release interleukin (IL)-2, which signals in an autocrine manner via the IL-2 receptor, whose α chain, called CD25, is expressed on T-cells upon antigen recognition and activation. IBD is characterized by elevated numbers of hiCD25+ cells, specifically affecting T-cells in CD and macrophages in UC [49]. Some intestinal CD4 T-cells from CD patients, but not UC patients, also express high levels of the activating natural killer group 2D receptor (NKG2D) [50], whose stimulation in combination with that of the TCR promotes the cytotoxic capacity of CD4 T-cells, plus the release of the pro-inflammatory cytokines tumor necrosis factor (TNF)-α, interferon (IFN)-γ, and IL-17A [50,51].

CD has usually been considered a type 1-driven disease, with the exacerbated production and activation of Th1 and Th17 cells and an elevated presence of their major cytokines IL-12, IL-23, IFN-γ, and IL-17. In contrast, UC has been designated as type 2-driven inflammation, linked to an elevated participation of Th2 and Th9 cells and their principal cytokines IL-13, IL-5, and IL-9 [52,53].

## 3. T Helper 1 (Th1) Cells

Th1 cells facilitate the eradication of intracellular pathogens, including parasites, protozoa, viruses, and intracellular bacteria, and intervene in cell-mediated immunity and delayed-type hypersensitivity reactions [54]. Th1 cells release IFN-γ and TNF-α, which stimulate innate immune cells, such as neutrophils and macrophages, and non-immune cells, such as epithelial cells and fibroblasts [55,56]. Th1 cells also release IFN-γ and IL-2 to recruit CD8 effector cytotoxic T-cells (CD8 CTL) [57].

Upon antigen recognition and the activation of a naïve CD4 T-cell, Th1 differentiation is mediated by the binding of IL-12 produced by the cognate APC. IL-12 induces T-cell expression of the master Th1 transcription factor T-box-containing protein (T-bet), encoded by the gene TBX21, and the cytokine IFN-γ, in both cases through a process dependent on STAT4 signaling stimulation [55,56,58]. T-bet increases the expression of IL-12 receptor subunit β2 (IL-12Rβ2), allowing synergistic IL-12 and STAT4 signaling to further increase IFN-γ generation [26,59,60].

In intestinal homeostasis, Th1 cells can prevent pathogen invasion and pathogen-derived antigens from mediating intestinal inflammation. Beside their direct antibacterial action, Th1 cells also ameliorate intestinal inflammation by secreting IL-2 and IL-10 to promote Treg stimulation. Moreover, Th1 cells can facilitate intestinal stem cell (ISC) proliferation and intraepithelial cell self-restoration by releasing low concentrations of TNF-α. Th1 cells thus constitute an immune barrier indispensable for intestinal homeostasis [61].

**Figure 2:**
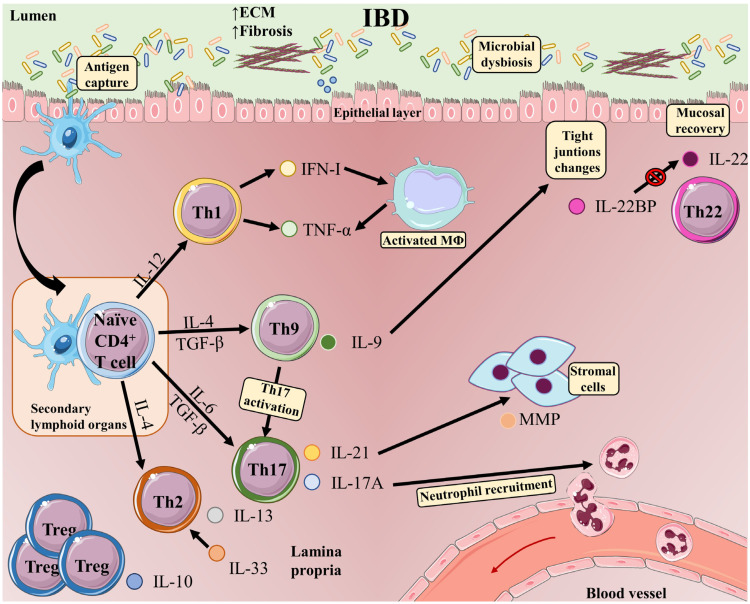
T-cell subsets and functions in the intestinal mucosa in inflammatory bowel disease. The development of IBD is induced by multiple phenomena occurring in the gastrointestinal tract: microbial dysbiosis, disruption of the mucus layer, dysregulation of epithelial tight junctions, defects in the number and function of Paneth cells, and increased intestinal permeability. These events massively increase bacterial exposure. In this context, antigen-bearing DCs capture antigens and migrate to secondary lymphoid organs, where they present antigens to naive T-cells. Once activated, CD4 T-cells undergo proliferation and differentiation into different effector T-cell subsets (Th1, Th9, Th17, and Th2 cells). Differentiated Th cells migrate back to the gut, where they carry out inflammatory functions, such as production of IFN-γ in the case of Th1 cells or IL-17A (which plays an important role in recruiting neutrophils to sites of active inflammation) and IL-21 (which induces MMP production by stromal cells) in the case of Th17 cells. Cytokines released by Th1 cells favor activation of macrophages, which release TNF-α and trigger epithelial-cell apoptosis. Th9 cells produce IL-9, which can act as a proinflammatory cytokine, activating Th17 cells. The presence of IL-9 is associated with alterations in the expression of tight junctions, and intestinal overproduction of IL-9 is likely to impair epithelial-barrier integrity and compromise tolerance to commensal bacteria, eventually progressing to inflammation. IL-33 is upregulated in UC patients and drives a Th2-like cytokine response. Elevated IL-33 production Th2 cells have also been reported in UC patients. Proinflammatory signals in IBD are counterbalanced by IL-10 produced by Tregs. IL-22 released by Th22 cells maintains intestinal epithelial barrier function. In inflamed intestinal tissue, CD4 T-cells are a major source of IL-22BP, which blocks IL-22 signaling.

A pathogenic role for Th1 cells has been described in the course of IBD (Figure 2). An excessive Th1 response has been observed in the inflamed mucosa and serum of IBD patients [62]. Classically, an exacerbated Th1 response has been linked to CD, whereas UC has been considered a Th2 cell-driven disease [63]. However, both UC and CD feature activated effector Th1 cells, suggesting that Th1 cells are implicated in the origin and development of mucosal inflammation in IBD [64]. 

The elevated levels of IL-12 and IL-18 detected in IBD support an involvement of exacerbated Th1 immune responses and intestinal inflammation in CD [65,66]. These two cytokines, which are produced by macrophages, stimulate the production of IFN-γ by Th1 cells, and the blockade of IL-12 or IL-18 reduces IFN-γ production [65,66]. Early CD also features increased mucosal levels of the typical Th1 cytokines IFN-γ and IL-21 [67]. 

Th1 differentiation and function depend on the proteins T-bet, IFN-γ, and TNF-α. Supporting the importance of Th1 differentiation in IBD, a lack of IFN-γ in CD4 T-cells prevents the development of dextran sulfate sodium (DSS)-induced colitis in mice [68]. In humans, the IBD-associated single nucleotide polymorphisms, rs1551398 and rs1551399, alter T-bet binding sites and predispose their carriers to increased mucosal inflammation [69]. The elevated levels of TNF-α and IFN-γ in the intestinal epithelium in IBD disturb intraepithelial cell functions. TNF-α promotes intraepithelial cell apoptosis and inflammation [70], whereas IFN-γ stimulates macrophages and neutrophils and promotes immune-cell recruitment by inducing the expression of adhesion molecules on intraepithelial cells [71]. The direct destruction of intraepithelial cells has been attributed to an epithelial cell adhesion molecule (EpCAM)-specific action of IFN-γ+ Th1 cells upon antigen presentation by DCs [72,73,74].

The evidence that Th1 cells play different roles in CD and UC includes the observation that Th1 cells isolated from the lamina propria of CD patients produce more IFN-γ than cells from UC patients or control individuals [75,76,77,78]. However, other authors did not detect these differences [79,80]. 

The transcription factor interferon regulatory factor 5 (IRF5) aggravates experimental colitis by increasing the CD4 T-cell expression of Th1- and Th17-related cytokines and reducing the expression of Th2-related cytokines [81]. Another regulator of transcription, among other functions, is the nuclear envelope protein lamin A/C, which promotes T-cell activation [82,83] and Th1 differentiation [84], inhibits Treg differentiation [85], and aggravates IBD in a cell adoptive transfer mouse model of colitis [86].

The specific binding of the integrin αEβ7 to E-cadherin on epithelial cells promotes the retention of Th1 in the intestinal mucosa [87]. Moreover, αEβ7+ Th1 cells express higher levels of IFN-γ and TNF-α than αEβ7- cells in the intestine of UC patients [88]. Finally, the inhibition of T-cell activation with a selective Ca2^+^ release-activated Ca2^+^ channel blocker inhibits IFN-γ production by organ culture biopsies from IBD patients [89]. In summary, Th1 cells influence the severity of intestinal inflammation.

## 4. T Helper 2 (Th2) Cells

Th2 cells participate in the elimination of extracellular microbes and intestinal helminths and support IgE-mediated B-cell responses by secreting IL-4, IL-5, IL-13, and IL-10 [90]. Th2 polarization is mediated by IL-4-ligation–dependent STAT6 signaling and the production of the Th2 master transcription factor GATA binding protein 3 (GATA-3) [91,92]. In addition to IL-4, Th2 cells produce the cytokines IL-5, IL-13, IL-21, and IL-25. Th2 cytokines prevent Th1 differentiation and promote the activation of macrophages [54,55]. Impaired Th2 responses are linked to allergies and asthma [93,94,95,96,97,98].

Oxazolone-induced colitis in mice involves a Th2 response featuring IL-5 and IL-4 production [99]. Another important Th2 cytokine is IL-33, which is elevated in UC patients and in mouse models of colitis induced with trinitrobenzenesulfonic acid (TNBS) or DSS. Moreover, IL-33 and the IL-33 receptor ST2 (suppression of tumorigenicity 2) are associated with IBD risk loci [53,100,101,102,103,104,105,106,107]. A lack of ST2 in mice diminishes colitis, whereas the administration of exogenous IL-33 aggravates the condition. These effects are associated with increased amounts of the Th2 cytokines IL-4, IL-5, and IL-13; major reductions in IL-17 and IFN- γ; damage to the epithelial barrier; and delayed wound recovery in the damaged colonic epithelium [53,100,101,102,105,106,107]. In contrast, IL-33 protects against intestinal inflammation by promoting the differentiation of forkhead box P3 (Foxp3)+ Tregs and innate lymphoid cells (ILCs) and by inducing the expression of amphiregulin [108,109].

Nevertheless, the treatment of UC with the anti-IL-13 monoclonal antibodies, tralokinumab and anrukinzumab, has not produced clinical benefits [110,111]. The levels of IL-36β, a member of the IL-1 cytokine family, are elevated in IBD patients, and IL-36β exacerbates DSS-induced colitis in mice by promoting Th2 responses in the lamina propria while reducing Foxp3+ Treg responses [112]. 

## 5. T Helper 9 (Th9) Cells

Th9 cells, like Th2 cells, intervene in the response to intestinal helminths [113] and have been linked to allergy and autoimmunity [114]. The differentiation of Th9 cells is induced by the concurrent action of IL-4 and transforming growth factor-beta (TGF-β). IL-4 binding to the IL-4 receptor triggers GATA3 transcription and the phosphorylation and dimerization of STAT6, promoting Th2 differentiation, whereas TGF-β activates FOXP3, inducing Treg differentiation [115,116]. In combination, IL-4 and TGF-β induce the production of IL-9 and the polarization of CD4 T-cells towards the Th9 phenotype [117,118,119]. Th9 differentiation depends on multiple transcription factors, including PU.1 and IRF4 [120,121]. Th9 differentiation can also be induced by other molecular combinations [122], such as IL-4 plus IL-1β [123]. Th9 cells are the main source of IL9, but also release IL-10 [118,124]. IL-9 can act as a proinflammatory cytokine, activating Th17 cells [125], and shares the same γ-chain receptor as IL-4, IL-2, and IL-15. IL-9 binding to its receptor activates janus kinase (JAK)1 and JAK3, which form dimers with STAT3, STAT5, or STAT1 [126,127,128].

The contribution of Th9 cells and their role in gut immunity have been demonstrated in several studies. Altered tissue integrity and continuous inflammation during flare-up episodes in UC are associated with IL-9 release by Th9 cells in the colon [129,130]. The presence of Th9-derived IL-9 is associated with alterations in the expression of tight junctions [131].

Several studies performed with IBD patient samples and mouse models have shown increased levels of IL-9 and Th9-related transcription factors [129,132,133,134]. Th9 cell numbers and activity are increased in the inflamed mucosa of UC patients [135]. The intestinal overproduction of IL-9 is likely to affect epithelial-barrier integrity and compromise tolerance to commensal bacteria, potentially progressing to inflammation [135].

Th2 and Th9 responses are interrelated, and elevated IL-9 and Th9 cell numbers, such as increased Th2 responses, are especially important in UC [136]. In the Th2-dominant oxazolone-induced colitis mouse model [3], IL-9 expression is increased throughout the intestinal tract, and the number of intestinal and splenic IL-9+ CD4+ T-cells is higher than in control mice [137]. A lack of IL-9 in the oxazolone-induced colitis model was found to reduce histological and disease symptoms and to enhance intestinal-barrier function [129]. Interestingly, in the TNBS-induced colitis mouse model, which features a potent Th1 response that resembles CD [3], mice lacking IL-9 show less severe inflammation and weight loss than wild-type mice. Moreover, IL-9-deficient mice in the TNBS-induced colitis model showed much less prominent goblet cell impairment, wound stimulation, and mononuclear cell deposition [130]. This discrepancy between models suggests that the role of Th9 and IL-9 in the development of IBD depends on the local microenvironment. This microenvironment is dominated by Th2 responses in the oxazolone-induced colitis model, provoking a more inflammatory Th9 response, and by Th1/Th17 responses in the TNBS-induced colitis model, promoting a tolerogenic-biased Th9 response [3,138]. The Th1/Th2 cytokine milieu of a third mouse model of colitis, induced with DSS, is considered to resemble both UC and CD [3]. These mice show elevated numbers of Th9 cells expressing PU.1 and CD3 markers, and IL-9 antibody-blockade was found to reduce disease symptoms and the presence of inflammatory mediators by reducing lymphocyte activity in the mouse intestinal lamia propria [139].

TNF-like factor (TL)1A and its receptor death receptor (DR)3 belong to the TNF and TNFR protein superfamilies. The attachment of APC-derived TL1A to lymphocyte DR3 provides co-stimulation to activated lymphocytes. DR3-dependent signaling modulates proliferative activity of and cytokine production by effector lymphocytes while also significantly impacting the generation and inhibitory capacity of Tregs [140]. Intestinal inflammation in chronic DSS-induced colitis is aggravated by elevated TL1A expression. In this model, TL1A may promote Th9 differentiation and IL-9 release by upregulating the expression of TGF-β, IL-4, and PU.1, suggesting a new target for IBD treatment [141]. In another study, inflamed tissue from UC patient intestine was found to contain elevated amounts of IL-9, IL-6, and IL-17A mRNA, and IL-9 mRNA levels correlated with the inflammation score [132]. The expression of TGF-β and IL-4, which potentiate Th9 cell differentiation [142], is increased in IBD patients and correlates with inflammation and disease symptoms [132]. 

IL-9-producing T-cells from UC patients show augmented expression of α4β7 integrin, which mediates the homing of Th9 cells to the intestine [143,144], and αEβ7 and α4β7-expressing T lymphocytes accumulate in UC-patient intestine. αEβ7 integrin binds to E-cadherin and MadCam in the intestine, promoting T-cell retention [144]. Th9 accumulation in UC patients can be abrogated by the blockade of the β7 subunit of integrin α4β7 and αEβ7 with the monoclonal antibody Etrolizumab [145].

In summary, mouse and human studies suggest that Th9 cells and their main cytokine IL-9 play a prominent role in IBD pathogenesis, especially in UC. Knowledge of how the recruitment and action of Th9 cells can be manipulated is necessary for improving therapeutic strategies.

## 6. T Helper 17 (Th17) Cells

Th17 cells protect the host from bacterial and fungal infections on mucosal surfaces but are also implicated in inflammatory and autoimmune diseases [146]. Th17 cells have thus been identified as pathogenic cells in relation to tissue inflammation and autoimmune disease [147,148,149]. However, it is becoming clear that Th17 cells also have a non-pathogenic phenotype with immune-modulatory functions [61,150,151,152,153].

Pathogenic and non-pathogenic Th17 cells can be polarized in vitro [154]. A combination of IL-6, IL-23, and IL-1β promotes pathogenic Th17 differentiation [155,156], whereas TGF-β1, in addition to IL-6, favors non-pathogenic Th17 cells [150,157,158,159]. IL-23 appears not to promote Th17  differentiation directly since naïve T-cells do not express the IL-23 receptor (IL-23R) in vitro, thus suggesting that IL-23 stabilizes the Th17 phenotype and promotes Th17 cell survival [156].

Pathogenic and non-pathogenic Th17 cells both express the transcription factor retinoic acid receptor-related orphan nuclear receptor gamma (RORγt) [160] in a STAT3-dependent manner [161] and produce IL-17 [61]; however, they have distinct genetic signatures, one contributing to immune injury and the other to immune homeostasis [154,162]. Pathogenic Th17 cells are characterized by the production of pro-inflammatory molecules, such as granulocyte-macrophage colony-stimulating factor (GM-CSF) and IL-23R, and by a low expression of immune-regulatory molecules, such as IL-10 and CD5 molecule like (CD5L). In contrast, non-pathogenic Th17 cells produce low amounts of GM-CSF and IL-23R and high amounts of IL-10 and CD5L, facilitating tissue homeostasis [150,151,156,163,164]. 

In settings of inflammation, Th17 cells produce IL-17A and IL-17F [160], members of the IL-17 family of proinflammatory cytokines, which run from IL-17A through F, with IL-17A frequently denoted as IL-17 [165]. IL-17 binds to a heterodimeric receptor (composed of IL-17RA and IL-17RC), which is expressed on many non-hematopoietic cells, including intestinal epithelial cells and on some activated T-cells [166]. The binding of IL-17 to its receptor regulates intestinal barrier function and the release of inflammatory chemokines and cytokines by target cells [167].

Other cytokines produced by Th17 cells include IL-22 and IL-21. At sites of inflammation, Th17 cells secrete several chemokines that promote the recruitment of neutrophils (chemokine (C-X-C motif) ligand (CXCL)1, CXCL2, CXCL5, and CXCL8) [168], as well as Tregs and more Th17 cells (chemokine receptor 6 (CCR6) and their ligands (C-C motif ligand (CCL20)) [165]. These Th17 cells also secrete granulopoiesis factors (granulocyte colony-stimulating factor (G-CSF)) and mediators of the acute phase response, including IL-6. IL-17 also stimulates the activity of matrix metalloproteinases (MMPs). Commensal bacteria promote IL-17 and IL-22 production, which in turn promote the production of barrier-protective cytokines and antimicrobial peptides [169,170].

Unlike Th1 and Th2 cells, Th17 cells show great plasticity and are able to differentiate into Th1, Tregs, and Tfh cells [61,171]. 

The binding of Th17-cell expressed CCR6 to the chemokine CCL20 recruits Th17 cells to the intestine, where they secrete IL-17, IL-21, IL-22, and TNF-α [172]. In homeostasis, Th17 cells control the proliferation and differentiation of lymphocytes, macrophages, and neutrophils; combat infection; and protect the integrity of the intestinal barrier [173,174]. However, alterations in Th17 cell number and functions can promote uncontrolled inflammation and mediate the development of IBD [30,61,171,172,175,176,177]. 

Th17 cells are more numerous in the peripheral blood of IBD patients, and several major Th17 cytokines, such as IL-17, IL-21, and IL-23, are abundant in the inflamed mucosa of these patients [178]. 

Compared with UC and healthy individuals, CD patients have elevated serum IL-17A and Th17 cell numbers in gut-draining lymph nodes [179]. Moreover, the numbers of IL-17-expressing cells are increased in the gut of patients with active CD and UC relative to the numbers in healthy individuals and patients with inactive CD or UC [180].

High levels of IL-17 and IL-21 promote the production of matrix MMPs by myofibroblasts, which results in the lysis of the extracellular matrix and epithelial cell damage. These cytokines also promote epithelial cells to release chemokines that stimulate inflammatory cell recruitment [181]. For example, IL-17 promotes IL-8 release by epithelial cells, stimulating the recruitment of neutrophils and Th17 cells to the inflamed tissue [175]. In line with these observations, a lack of the IL-17 receptor (IL-17R) in mice protects against TNBS-induced colitis [182], and a lack of IL-17F confers resistance to DSS-induced colitis [183]. However, the absence of IL-17 aggravates DSS-induced colitis, indicating that IL-17 also has beneficial effects [184]. IL-21 released by Th17 cells acts in an autocrine manner to promote their differentiation and the production of IL-17. Th17 responses are also supported by autocrine production of IL-23 [185]. Increased levels of IL-21 have been found in mice with chronic DSS-induced and TNBS-induced colitis, and IL-21 blockade by the addition of neutralizing IL-21R fusion proteins to DSS-treated mice mitigates colitis and inhibits the release of the main Th17 cytokines [186]. IL-21 enhances Th1 responses and IFNγ production by both Th1 and NK cells [187]. 

Th17 cells release TNF-α, which binds to the receptors TNFR-1 and TNFR-2 [188] and enhances IBD [189]. Th17 cells in the gut of CD patients can secrete IL-17 and IFNγ together, a finding confirmed by treating Th17 cells in vitro with the pro-Th1 cytokine IL-12 [190]. The transition of Th17 cells to the Th1 phenotype has also been reported in experimental mouse models, including of IBD [191]. Surprisingly, the blockade of IL-17A or IL-17R with the antibodies Secukinumab or Brodalumab in patients with moderate to severe CD generated more serious adverse events in the treatment group than in patients receiving the placebo [192,193]. Increased disease scores and symptoms were also observed in DSS-induced colitis upon the blockade of IL-17A [194] or in IL-17 KO mice [169]. Colitis-like disease is also promoted by the transfer of Th cells from mice lacking IL-17A or IL-17RA into mice that are deficient for recombination activating gene (RAG)1 [167].

Similarly to the situation described above for Th9 cells, mice genetically deficient for IL-17R or treated with IL-17R-Ig fusion protein develop less severe IBD upon treatment with TNBS [182], suggesting that the protective role of IL-17 may depend on the specific colitis model and the local microenvironmental inflammatory conditions. 

A possible explanation for these conflicting results is that the inhibition of the Th17 response can potentiate the more proinflammatory Th1 phenotype, as reported in a study that found increased intestinal Ifng mRNA and Th1 polarization in the absence of IL-17A, reflecting the ability of IL-17 to diminish the expression of IFN-γ and thus potentially enhance the stability of the Th17 phenotype by limiting Th1 differentiation [167]. Alternatively, the protective function of IL-17A may be related to its ability to regulate the epithelial barrier function and gut homeostasis [195]. Supporting this, IL-17 antibody blockade enhances the permeability of the intestinal epithelial barrier in DSS-induced colitis in mice [196]. In both cases, the increase in permeability correlated with changes in epithelial tight junction gene expression and in occludin positioning within the damaged epithelial layer [195,196].

IL-17 also modulates anti-microbial peptide release, potentially modulating microbial populations within the gut in IBD [195]. IL-17, in concert with fibroblast growth factor 2, also controls both epithelial barrier maintenance and bacterial homeostasis in the intestine [169]. Together, these data indicate a proinflammatory effect of Th17 in concert with a role in maintaining a healthy epithelial barrier and an optimal bacterial balance. 

IL-23 promotes the expansion of pathogenic Th17 cells by maintaining Th17 signature genes, upregulating effector genes, such as IL17A, IL17F, or IL22, or repressing suppressive factors. Moreover, IL17 and IL23 signaling promote pro-inflammatory molecules such as TNF, IFNγ, IL22, lymphotoxin, and IL1β [176]. Several mouse models of colitis have shown an augmented production of IL23 [197,198,199,200]. In patients, treatment with selective IL23 inhibitors promotes better response rates in the cohort of CD patients that failed prior anti-TNF therapy (reviewed in [176]), and IL23 targeting in UC patient, is safe and effective and promote and sustain clinical remission, low inflammation, mucosal healing, and an improved quality of life (reviewed in [201]). These experiments indicate the importance of the IL23/IL17 axis in mucosal inflammation.

## 7. T Helper 22 (Th22) Cells

Th22 cells protect against tissue damage and bacterial infection by producing the IL-10 family member IL-22 [202,203,204]. Th22 cells also produce IL-13, fibroblast growth factor, chemokines, and TNFα. IL-22 is also secreted by Th1 and Th17 cells, but Th22 cells are able to secrete IL-22 without producing IFN-γ or IL-17 [54,205]. IL-22 is also secreted by NKs, γδT cells, ILC3s, and some nonlymphoid cells [206]. Th22 cells express the chemokine receptors CCR10, CCR6, and CCR4, and their differentiation is promoted by the activation of STAT3 and the aryl hydrocarbon receptor (AHR) by IL-6, TNF-α, and IL-1β and is diminished by TGF-β [207,208]. 

IL-22 enhances innate immunity by modulating cell differentiation, chemokine secretion, and antimicrobial peptide (AMP) secretion [209,210,211]. In the intestinal epithelium, IL-22 promotes the secretion of AMPs, such as β defensins and lipocalin 2 and the mucin proteins MUC1 and MUC3 [212]. IL-22 can also promote the secretion by human colonic myofibroblasts of the anti-inflammatory factor IL-11 and inflammatory molecules, such as IL-6 and CXCL chemokines [213].

In healthy individuals, IL-22 is released mainly in the gastrointestinal tract, where it favors mucosal recovery [214,215]. This beneficial effect is mediated by the binding of IL-22 to the receptor IL-22R, whose expression is mostly limited to epithelial cells [214]. 

IL-22 maintains intestinal epithelial barrier function by promoting the release of antimicrobial peptides [202] and mucins [215], as well as by facilitating intestinal epithelial cell survival and proliferation [214]. IL-22 can increase the production of anti-inflammatory factors, such as IL-11, that also protect epithelial barrier function [216]. 

However, elevated levels of IL-22 can be detrimental [206], enhancing the production of inflammatory mediators, such as IL-6 and CXCL chemokines by human colonic myofibroblasts [217]. IL-22 modulates neutrophil recruitment to the colon by controlling the expression of neutrophil-active CXC-family chemokines in ulcerative colitis; by this mechanism, the augmented expression of IL-22 is associated with treatment resistance to an anti-IL-12/23 p40 subunit monoclonal antibody [218]. 

IL-22 is secreted at low levels, and is mostly maintained in a biologically inactive state through the action of IL-22 binding protein (IL-22BP, also known as IL-22RA2), produced by intestinal DCs and macrophages in the gut lamina propria and secondary lymphoid structures [219,220,221,222,223]. In inflamed intestinal tissue, the main producers of IL-22BP are CD4 T-cells [217,220]. IL-22BP is a soluble receptor homolog that attaches to IL-22 with greater affinity than IL-22R, preventing IL-22 from binding to its receptor and thereby blocking IL-22 signaling [224,225]. Elevated levels of IL-22 and IL-22BP mRNA and protein have been detected in inflamed tissue from CD and UC patients [220,226,227]. Consistent with these findings, the IL-22-associated protection against DSS-induced colitis is increased in IL-22BP deficient rats [228], and IL-22BP aggravates T-cell-mediated colitis in mice [220]. IL-22BP expression is reduced in infectious colitis but not in inflamed tissues in IBD, indicating potential pathophysiological significance for IL-22BP-dependent alterations in IL-22 bioactivity [220,228].These responses may vary between patients and differ according to the extent of histological damage. For example, CD patients with granuloma are reported to have increased frequencies of IL-22+ and IL-22+ IFN-γ+ cells in colonic tissue [229].

Results from experiments in mice thus seem to indicate that high levels of IL-22BP in IBD can provoke inflammation by interrupting IL-22-mediated mucosal healing [220]. Supporting this, gut CD4 T-cells from anti-TNF-α-treated IBD patients show lower amounts of IL-22BP but still express IL-22 [220] and may even up-regulate IL-22 generation [230].

## 8. Regulatory T-Cells (Treg)

Treg cells suppress immune responses and maintain peripheral tolerance and immune homeostasis [231]. Tregs are divided into thymic-derived Tregs, also called natural Treg cells (nTregs) [232], and post-thymic maturation peripheral Tregs (pTregs) [233,234,235,236,237]. Tregs induced in vitro by the addition of TGF-β and IL-2 to naïve CD4 T-cells are called inducible Tregs (iTregs) [237,238].

Tregs are characterized by the secretion of the inhibitory cytokines IL-10, IL-35, and TGF-β, and the expression of the transcription factor Foxp3, which mediates Treg development, lineage commitment, and regulatory functions [55]. Another marker of nTregs and pTregs is the IL-2 receptor α chain CD25 [55]. 

nTregs are positively selected in the thymus by the intermediate affinity of the TCR for self-peptides/MHC [232], whereas T-cells with a high-affinity TCR antigen are eliminated and those with low-affinity differentiate into naïve T-cells [239]. In humans, nTreg development seems to also depend on IL-2 and/or IL-15 [240,241,242].

In the thymus, a restricted number of autoreactive CD4 T-cells differentiate into nTregs, in a process called agonist selection that guarantees central tolerance to self-antigens, thus avoiding autoimmunity [236,243,244]. nTregs are already in an antigen-primed or antigen-activated state in the thymus [238].

pTregs differentiate from conventional CD4 T-cells in the periphery under tolerogenic conditions in secondary lymphoid tissues, in particular intestinal draining lymph nodes, upon the recognition of an antigen presented by an APC [245,246,247]. pTreg differentiation requires the sustained expression of FOXP3 and is dependent on high levels of TGF-β, an absence of proinflammatory cytokines [54], and the activation of naïve CD4 T-cells upon recognition of mainly exogenous antigens [248,249,250]. pTreg differentiation is also facilitated by vitamin-A derived retinoic acid [251,252,253]. 

pTregs are classified as central, effector, and tissue-resident pTregs [254]. Central pTregs are considered naïve and in mice are characterized by the expression of the markers CD62Lhigh CCR7+ or CD45RAhigh CD25low. Central pTregs are the main Treg type in the circulation and in secondary lymphoid organs. The marker profile of effector Tregs, also called effector memory or activated Tregs, is CD62Llow, CCR7low, CD44hi killer cell lectin like receptor G1 (KLRG1)+, CD103+, or CD25RAlow CD25hi. Effector memory pTregs are less frequent than central pTregs and are similar to conventional activated CD4 T-cells that have had recent contact with an antigen. Tissue-resident pTregs found In non-lymphoid tissues, such as the colon, and under steady state conditions account for most pTregs in the intestine [254].

Tregs are activated at much lower antigen/MHC concentrations than naïve T-cells, ensuring Treg-dependent self-tolerance [255]. Tregs are a frequent immune cell population in the intestine, where they limit inflammatory CD4 T-cells [256,257] and maintain immune homeostasis through several mechanisms [258]. FoxP3+ Tregs, especially effector Tregs, are constantly proliferating under steady state conditions, likely as a consequence of identifying self-antigens and antigens derived from commensal microbes [259,260].

The suppressor activity of Tregs is mostly mediated by cell-contact–dependent and humoral-factor–mediated mechanisms. These mechanisms include IL-2 scavenging; the secretion of regulatory cytokines, such as IL-10, [261], IL-35 [262], and TGF-β [263,264]; the surface expression of inhibitory molecules, such as CTLA-4 (cytotoxic lymphocyte antigen 4) and PD-1 (programmed cell death 1), TIGIT (T-cell immunoreceptor with immunoglobulin and immunoreceptor tyrosine-based inhibition motif domains), CD39, and CD73 [265,266]; cytolysis; and metabolic control [238]. Tregs also promote tissue through the release of growth factor amphiregulin [267].

Treg numbers are increased in the inflamed tissue of IBD patients [268]. This increase may be the result of a compensatory mechanism to control the exacerbated proinflammatory immune response, but would seem to imply that Tregs are inefficient at suppression, since transient FOXP3 expression has been observed in human activated non-regulatory CD4 T-cells [253]. Recent single-cell RNA sequencing (scRNAseq) studies have provided a more detailed picture of the cell populations, including Tregs, in the healthy [269] and inflamed tissue of IBD patients and mice [48,270,271,272,273,274,275,276,277]. The inflamed epithelium of CD patients contains depleted numbers of Tregs, CD8 T-cells, γδT cells, and Tfh cells and elevated numbers of activated Th17 cells, as revealed by scRNAseq and multi-parameter flow cytometry or mass cytometry experiments [271]. scRNAseq has also revealed the persistence and expansion of CTLA-4+ Tregs in patients with checkpoint inhibitor-induced colitis [278]. In another study, inflamed tissue from UC patients was found to contain increased numbers of Tregs expressing FOXP3 and basic leucine zipper ATF-like transcription factor (BATF) and IL1B/LYZ+ myeloid cells [279]. These contradictory results illustrate that Treg changes remain unclear, and hint at a heterogeneous response among different Treg cell subsets in IBD. For example, inflamed tissue from CD patients contains increased numbers of RORγt+FOXP3+ Tregs, which secrete IL-17 and IFNγ while maintaining their suppressive function [280]. A similar population has been detected in UC patients [277]. It would thus seem that, although the lineage stability of Tregs allows them to maintain suppressive capacity and FoxP3 production despite exposure to inflammatory stimuli [281], Tregs can alter their phenotype by expressing transcription factors and chemokine receptors without producing inflammatory cytokines, helping them to arrive at the inflammation site where they can exert their suppressive effect on target T effector cells [282]. Through this mechanism, Tregs can acquire phenotypes similar to Th1 [283,284], Th2 [285,286,287], Th17 [288,289,290], or Tfh cells [291,292,293].

In the intestine, Tregs can acquire several phenotypes expressing varying levels of GATA3, Helios, and RORγt. GATA3+Helios+ Tregs seem to have a thymic origin and react to the alarmin IL-33 produced in response to tissue damage, reducing tissue injury in colitis [294]. RORγt+Helios− Tregs, produced in response to intestinal microbiota, are considered pTregs and play a protective role in severe gut inflammation [289,290]. RORγt−Helios− Tregs are more abundant in the small intestine and participate in the amelioration of allergic responses to food antigens [295]. These observations indicate that Tregs are highly versatile cells that adapt to their environment in order to better contribute to tissue homeostasis. There is some interest in developing therapies to boost Treg cell number and function and thereby reduce intestinal inflammation in IBD [254,296,297]. 

Other CD4 T-cell subsets include Foxp3- type 1 regulatory T (Tr1) cells, which secrete the suppressive cytokines IL-10 and TGF β [26,298], and Tfh cells, which are a specialized CD4 T-cell subset involved in the induction and differentiation of B cells into plasma cells and memory cells [299,300,301], cell subsets whose role in IBD has recently been reviewed [26,254,302,303].

## 9. Conclusions

Current IBD therapy is making great strides in improving patient quality of life. However, the high phenotypic variability of these patients means that, in many cases, treatments fall short of their objectives or simply do not work. Recent technological advances have contributed to a more detailed knowledge of the cell types involved in the pathophysiology of IBD, revealing the wide range of different phenotypic T-cell subsets and plasticity between them. Understanding the mechanisms that control changes between these phenotypes and determine the disease-promoting or disease-alleviating behaviors of these different cell subsets could lead to more specific therapies for each patient and each stage of the disease.

## Data Availability

No new data were created in this study. All the data reported in this review were found in original articles cited in the text.

## Abbreviations

AHR	Aryl hydrocarbon receptor
AMP	Antimicrobial peptide
APC	Antigen presenting cell
BATF	Basic leucine zipper ATF-like transcription factor
CCL	Chemokine (C-C motif) ligand
CCR	C-C motif chemokine receptor
CD	Crohn’s disease
CD5L	CD5 molecule like
CTL	Cytotoxic T cells
CTLA-4	Cytotoxic lymphocyte antigen 4
CXCL	Chemokine (C-X-C motif) ligand
DAMPs	Damage-associated molecular patterns
DCs	Dendritic cells
DR	Death receptor
DSS	Dextran sodium sulfate
EpCAM	Epithelial cell adhesion molecule
Foxp3	Forkhead box P3
GATA-3	GATA binding protein 3
G-CSF	Granulocyte colony-stimulating factor
GM-CSF	Granulocyte-macrophage colony-stimulating factor
IBD	Inflammatory bowel disease
IEC	Intestinal epithelial cells
IFN	Interferon
Ig	Immunoglobulin
ILCs	Innate lymphoid cells
IL	Interleukin
IL-12Rβ2	IL-12 receptor subunit β2
IL-22BP	IL-22 binding protein
IRF	Interferon regulatory factor
ISC	Intestinal stem cell
JAK	Activates janus kinase
KLRG1	Killer cell lectin like receptor G1
KO	Knockout
MHC	Major histocompatibility complex
MMPs	Matrix metalloproteinases
NK	Natural killer
NKG2D	Natural killer group 2D receptor
NLR	Nod-like receptors
PAMPs	Pathogen-associated molecular patterns
PD-1	Programmed cell death 1
PRRs	Pattern recognition receptors
RAG	Recombination activating gene
RORγt	Retinoic acid receptor-related orphan receptor gamma t
scRNA-seq	Single cell RNA sequencing
STAT	Signal transducer and activator of transcription
T-bet	T-box-containing protein
TCR	T cell antigen receptor
Tfh	T follicular helper
TGF	Transforming growth factor
Th	T helper
TIGIT	T cell immunoreceptor with immunoglobulin and immunoreceptor tyrosine-based inhibition motif domains
TL	TNF-like factor
TL1A	TNF-like ligand 1 A
TLRs	Toll-like receptors
TNBS	2,4,6-trinitrobenzene sulfonic acid
TNF	Tumor necrosis factor
Treg	T regulatory
Tr1	T regulatory type 1
UC	Ulcerative colitis
